# Endoscopic retrograde cholangiopancreatography and endoscopic cystogastrostomy in very young children (aged <5 years): Feasibility, success, and safety

**DOI:** 10.1002/deo2.70085

**Published:** 2025-02-23

**Authors:** Ujjal Poddar, Arghya Samanta, Samir Mohindra, Vijay Datta Upadhyaya, Basant Kumar, Anshu Srivastava, Moinak Sen Sarma, Surender Kumar Yachha

**Affiliations:** ^1^ Department of Pediatric Gastroenterology Sanjay Gandhi Postgraduate Institute of Medical Sciences Lucknow India; ^2^ Department of Gastroenterology Sanjay Gandhi Postgraduate Institute of Medical Sciences Lucknow India; ^3^ Department of Pediatric Surgical Superspecialties Sanjay Gandhi Postgraduate Institute of Medical Sciences Lucknow India

**Keywords:** hepato‐biliary, pancreatic diseases, pediatric, pediatric endoscopy, therapeutic endoscopy

## Abstract

**Objectives:**

Paucity of data and concerns about potential lower effectiveness and more adverse events limit the use of endoscopic retrograde cholangiopancreatography (ERCP) and endoscopic cystogastrostomy in younger children even in high‐volume centers. We retrospectively analyzed indications, success rates, and adverse events of all the children (<18 years) who underwent ERCP and endoscopic cystogastrostomy between January 2010 to May 2024 at our center.

**Methods:**

Data, including patient demographics, indications for the procedure, technical details, and adverse events, were collected from our prospectively kept database and compared according to age groups (<1 year, 1–5 years, 5–10 years, and 10–18 years).

**Results:**

A total of 286 ERCP (273 therapeutic and 13 diagnostic) and 57 endoscopic cystogastrostomy were performed in 222 (138 boys) and 55 children (32 boys), respectively, during the study period with 20% ERCP procedures in under‐five children. In children <5 years, the majority of the ERCPs were for biliary diseases (87%), while pancreatic duct procedures (39.5%) were done in higher numbers in children >5 years. For biliary ERCP, choledochal cyst (15, 33%) was the most common etiology in under‐five children and choledocholithiasis (60, 34%) in children >5 years. Cannulation and technical success rates were 95% and 92%, respectively with no significant difference across age groups. Adverse events were noted in 36 (16%) with post‐ERCP pancreatitis (8%) being the most common. All adverse events were managed conservatively with no mortality.

**Conclusion:**

ERCP can safely be performed in all children, including those under five with various hepato‐pancreato‐biliary diseases with high technical success rates.

## INTRODUCTION

Endoscopic retrograde cholangiopancreatography (ERCP) has made quantum leaps in technique and clinical application since its introduction in 1968.^1^ It has evolved from diagnostic purpose to primarily a therapeutic procedure, and is currently a well‐established method for the treatment of hepatobiliary and pancreatic diseases in adults. Although, Dr. J Waye reported a case of ERCP in an infant way back in 1976,^2^ its role in the pediatric population has expanded gradually over time.^3^, ^4^, ^5^, ^6^ However, most studies on ERCP in the pediatric population included older children from developed countries with limited reports from low‐ and middle‐income countries.^7^, ^8^, ^9^, ^10^, ^11^, ^12^ The feasibility, utility, and safety of ERCP in younger children, especially in the under‐five age group has not been highlighted so far. We, therefore, aimed to evaluate the indications, utility, and safety of ERCP in under‐five children with hepato‐pancreato‐biliary disorders.

## METHODS

### Study populations

Prospectively kept a database of all the children aged <18 years of age, who underwent ERCP due to various hepato‐pancreatico‐biliary disorders between January 2010 and May 2024 in our center was analyzed retrospectively. Data, including patient demographics, indications, and technical details of the procedure and adverse events if any, were retrieved from our electronic database (hospital information system) and manual records. The data were compared according to age groups of children (<1 year, 1–5 years, 5–10 years, and 10–18 years).

### Procedure and equipment

All the procedures were done on an inpatient basis after taking informed written consent from either parent. All procedures were performed by experienced pediatric gastroenterologists (more than 5 years of experience performing ERCP, and endoscopic cystogastrostomy) under moderate sedation by midazolam and ketamine in the endoscopy suite under fluoroscopic control (Axiom Iconos R 200; Siemens). A standard adult duodenoscope (Olympus TJF 160VR; Olympus) with an outer diameter of 13.5 mm and a working channel diameter of 4.2 mm was used for all procedures in children >12.5 kg. In smaller children (1–3 years) with a body weight <12.5 kg, a diagnostic duodenoscope (Olympus JF‐140R) with an outer diameter of 11 mm and working channel diameter of 3.2 mm and for infants with suspected biliary atresia, pediatric duodenoscope (PJF‐160R; Olympus) with an outer diameter of 7.5 mm and working channel diameter of 2 mm were used. Endoscopic cystogastrostomy was done using the standard technique with an adult duodenoscope. The techniques and accessories used were similar to those routinely used in adult patients. Pancreatic duct stones of >5 mm were first fragmented with extracorporeal shock wave lithotripsy (ESWL) followed by endoscopic removal.^13^ All the patients were hydrated with intravenous fluids during and after the procedure, and rectal non‐steroidal anti‐inflammatory drugs were administered 1 h prior to the procedure to patients deemed to be at high risk of post‐ERCP pancreatitis (PEP).^14^ Radiation exposure was kept to a minimum by limiting fluoroscopy time. The patients were observed for 24 h or longer after the ERCP for assessment of any adverse events as an inpatient. If the child developed pain abdomen any time after the procedure, they were evaluated for the cause of pain and started on symptomatic treatment (nil per orally, intravenous fluids, and analgesics). Adverse events were defined according to the criteria developed by the American Society of Gastrointestinal Endoscopy.^15^ Adverse events such as PEP were defined as new or worsened abdominal pain for more than 24 h after endoscopy with raised serum amylase or lipase levels to >3 times of upper limit of normal which required prolongation of planned hospitalization for more than 2 days.^16^ The clinical severity of PEP was classified as per the revised Atlanta classification, 2012.^17^ All adverse events were managed as per standard protocol.

### Outcomes parameters

#### Endoscopic retrograde cholangiopancreatography

Cannulation success was defined as successful deep cannulation of the desired duct, whereas technical success was defined as successful deep cannulation along with completion of the planned therapeutic procedure.

#### Endoscopic cystogastrostomy

Cannulation success rate was defined as cannulation and passing of guidewire into cyst cavity while technical success rate was defined as completion of the planned therapeutic procedure.

### Ethics

The study was performed in a manner to conform with the Helsinki Declaration of 1975, as revised in 2000 and 2008, concerning Human and Animal Rights. Informed consent was obtained from either parent before all procedures. Institutional ethical clearance was taken from the institutional ethical board (IEC 2024‐163‐IP‐EXP‐60).

### Statistical analysis

IBM‐SPSS statistical software version 22 (SPSS) was used to perform the statistical analysis. A *p*‐value of <0.05 was considered statistically significant. Continuous variables are expressed as median with interquartile range (IQR) and discrete variables as proportions. Comparisons of continuous variables between two groups were performed using the independent *t*‐test and Mann–Whitney *U*‐test as per normality of data distribution. Paired *t*‐test was used to compare two variables within the group. Variables with skewed distribution were compared using the Kruskal–Wallis test followed by the Mann–Whitney test with adjusted *p*‐values.

## RESULTS

A total of 286 ERCP (273 therapeutic and 13 diagnostic) and 57 endoscopic cystogastrostomy were performed on 222 (138 boys) and 55 children (32 boys) respectively at our center between January 2010 and May 2024. Among those undergoing ERCP, 45 (20%) were under‐five children while among those undergoing endoscopic cystogastrostomy, five (9%) were under‐five children.

**FIGURE 1:**
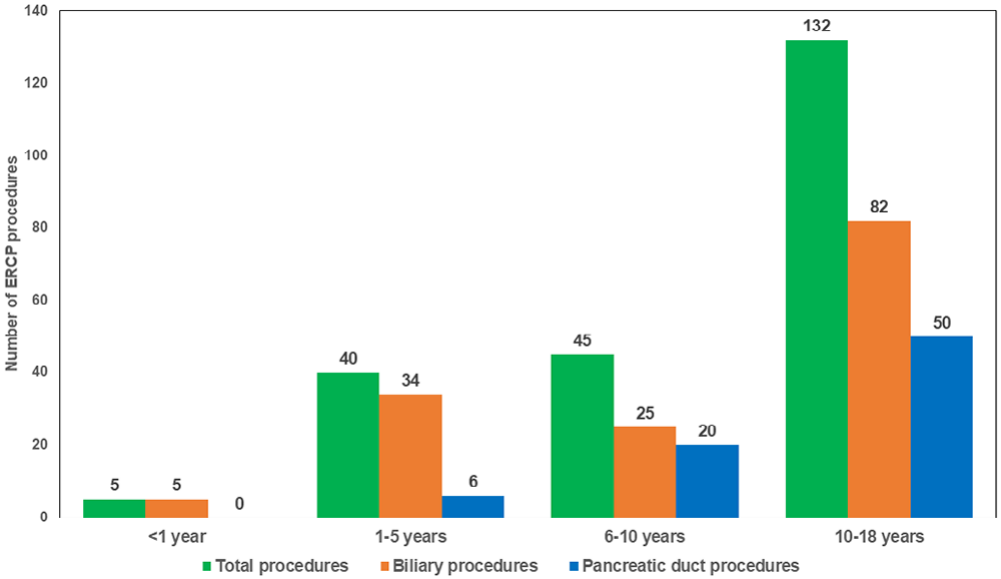
Age group‐wise distribution of all endoscopic retrograde cholangiopancreatography (ERCP) procedures.

The median age of 222 children undergoing ERCP was 9.4 years (IQR: 4.00–13.75) with a range from 3 months to 17 years. Out of these 286 ERCP procedures, 62 (20%) were performed in 45 under‐five children; 57 (92%) were therapeutic and five (8%) diagnostic (Table 1). Overall, 38 (14%) children underwent more than one session of ERCP and 13 of them aged less than 5 years. Age group‐wise distribution of patients and indications of ERCP are summarized in Table 1 and Figure 1. The number of children undergoing ERCP procedures increased with age. Overall, biliary diseases (146, 66%) were the most common indication for ERCP, followed by pancreatic duct disorders (76, 34%). In infants, all the ERCP procedures were diagnostic to exclude biliary atresia while in children older than one year of age, the majority of the procedures were therapeutic (98% vs. diagnostic 2%, *p* < 0.001).

**TABLE 1:** Indications of endoscopic retrograde cholangiopancreatography (ERCP) procedures in children across various age groups.

Indications	<1 year *n* (%)	1–5 years *n* (%)	5–10 years *n* (%)	10–18 years *n* (%)	All age groups *n* (%)	*p*‐value
Total patients	5 (2)	40 (18)	45 (20)	132 (60)	222	0.063
Median age in years, (IQR)	0.3 (0.3–0.4)	3.1 (2–4.7)	7 (5.8–9)	13.5 (12–15)	9.4 years (IQR 4–13.75)	–
Gender, Male	3 (60%)	30 (53%)	34 (55%)	71 (44%)	138 (62%)	0.62
Median body weight in kg (IQR)	4 (3.5–4.3)	12.6 (11–14.5)	23.8 (20–27.5)	38.5 (32–43)	34.5 (19–40.5)	–
Total procedures	5 (1.7)	57 (20)	62 (21.7)	162 (56.6)	286	0.061
**Indications**						
**• Biliary duct procedures**	5 (100)	34 (85)	25 (55)	82 (62)	146 (66)	0.04
**Therapeutic**						
Isolated choledocholithiasis	0 (0)	8 (23.5)	10 (40)	50 (60)	68 (46.5)	0.043
Choledochal cyst	0 (0)	15 (44)	4 (16)	2 (2.3)	21 (14)	0.004
Biliary ascariasis	0 (0)	1 (3)	0 (0)	0 (0)	1 (0.7)	0.067
Bile duct leak^*^	0 (0)	4 (12)	2 (8)	2 (2.3)	8 (5.4)	0.42
common bile duct stricture	0 (0)	2 (6)	1 (4)	16 (19)	19 (13)	0.054
Liver abscess with common bile duct fistula	0 (0)	1 (3)	2 (8)	4 (4.8)	7 (4.8)	0.07
Biliary pancreatitis	0 (0)	3 (9)	1 (4)	5 (6)	9 (6)	0.72
**Diagnostic**						
Suspected biliary atresia	5 (100)	0 (0)	0 (0)	0 (0)	5 (3.4)	–
Sclerosing cholangitis	0 (0)	0 (0)	5 (20)	3 (3.5)	8 (5.5)	0.12
**• Pancreatic duct procedures**	0	6 (15)	20 (44)	50 (38)	76 (34)	0.03
Chronic pancreatitis with MPD stricture	0	5(84)	17 (85)	42 (84)	64 (84)	0.65
Pancreatic duct leak (post‐trauma)	0	1(16)	3 (15)	8 (16)	12 (16)	0.87

All the procedural outcomes were analyzed based on based on patient‐based numbers.

^#^ *p*‐value is calculated for particular therapeutic procedures across age groups 1–5, 5–10, and 10–18 years as a very less number of procedures done in children <1 year.

Abbreviations: kg, kilogram; IQR, interquartile range.

* (Post‐traumatic 6 and post‐cholecystectomy 7).

^**^WON: walled‐off necrosis.

In children younger than five years, the majority of the ERCP procedures were for biliary diseases (85%), while in older children, pancreatic duct procedures (39.5%) were done more frequently (Table 1 and Figure 1). Among the biliary diseases, choledochal cyst (37.5%) was the commonest etiology, followed by choledocholithiasis (20%), and bile leak (10%) in children between 1 and 5 years. On the contrary, among the children of 5–10 and 10–18 years age groups, choledocholithiasis (40% and 60%, respectively) was the commonest etiology, with a choledochal cyst in a minority (16% and 2.3%, respectively) of patients (Table 1). Among the pancreatic duct procedures, chronic pancreatitis with pancreatic duct stricture (84%) was the most common indication, followed by post‐traumatic pancreatic duct leak (16%; Table 1).

Details of all the therapeutic interventions are summarized in Table 2. Among the biliary procedures, sphincterotomy was the most common procedure, followed by balloon sweeping, stent placement, and stone extraction. Among the pancreatic duct procedures, sphincterotomy was again the most common procedure, followed by balloon dilatation, stent placement, and stone extraction. The frequency of the procedures was similar in children below and above five years.

**TABLE 2:** Therapeutic interventions done in all endoscopic retrograde cholangiopancreatography (ERCP) procedures.

Therapeutic procedures	<1 year *n* (%)	1–5 years *n* (%)	5–10 years *n* (%)	10–18 years *n* (%)	All age groups, *n* (%)	*p*‐value^#^
Total patients	5	40	45	132	222	–
Total procedures	5	57	62	162	286	–
Canulation success rate	5 (100)	36 (90)	42 (93)	127 (96)	210 (94.5)	0.68
Technical success rate	5 (100)	35 (87.5)	40 (89)	124 (94%)	204 (92)	0.63
Biliary procedures	5	34	25	82	146	
Conventional sphincterotomy	5 (100)	28 (82)	18 (72)	50 (61)	101 (69)	0.87
Pre‐cut sphincterotomy	0 (0)	1 (3)	1 (4)	2 (2.5)	4 (2.7)	0.37
Balloon dilatation	0 (0)	4 (12)	4 (16)	18 (22)	26 (18)	0.35
Cholangiogram	5 (100)	25 (73.5)	21 (84)	55 (67)	106 (72.6)	0.67
Balloon sweeping	0 (0)	27 (79)	19 (76)	21 (25)	67 (46)	0.072
Plastic stent placement						
5 Fr	0 (0)	4 (12)	1 (4)	0 (0)	5 (3.5)	0.17
7 Fr	0 (0)	24 (70)	16 (64)	42 (51)	82 (56)	0.72
10 Fr	0 (0)	0 (0)	0 (0)	11 (13)	11 (7.5)	0.15
Stent removal	0 (0)	9 (26.5)	5 (2)	24 (30)	38 (26)	0.82
Pancreatic duct procedures	0	6	20	50	76	
Conventional sphincterotomy	0 (0)	6 (100)	15 (75)	38 (76)	59 (77.6)	0.13
Pre‐cut sphincterotomy	0 (0)	0 (0)	0 (0)	2 (4)	2 (2.5)	0.067
Major papilla cannulation	0 (0)	5 (84)	17 (84.6)	45 (90)	67 (88)	0.71
Minor papilla cannulation	0 (0)	1 (16)	2 (10)	2 (4)	5 (6.5)	0.75
Balloon dilatation	0 (0)	6 (100)	15 (75)	38 (76)	59 (77)	0.83
Pancreatogram	0 (0)	5 (84)	18 (90)	47 (94)	70 (92)	0.85
Stone retrieval by balloon sweeping	0 (0)	3 (50)	3 (15)	12 (24)	18 (23)	0.14
Plastic stent placement						
5 Fr	0 (0)	4 (67)	2 (20)	0 (0)	6 (8)	0.002
7 Fr	0 (0)	2 (33)	11 (55)	22 (44)	35 (46)	0.43
10 Fr	0 (0)	0 (0)	0 (0)	15 (30)	15 (20)	0.004
Stent removal	0 (0)	0 (0)	5 (25)	10 (20)	15 (20)	0.26

All the procedural outcomes were analyzed based on based on patient‐based numbers.

Abbreviation: Fr, French.

^#^ *p*‐value is calculated for particular therapeutic procedures across age groups 1–5, 5–10, and 10–18 years as a very less number of procedures done in children <1 year.

**FIGURE 2:**
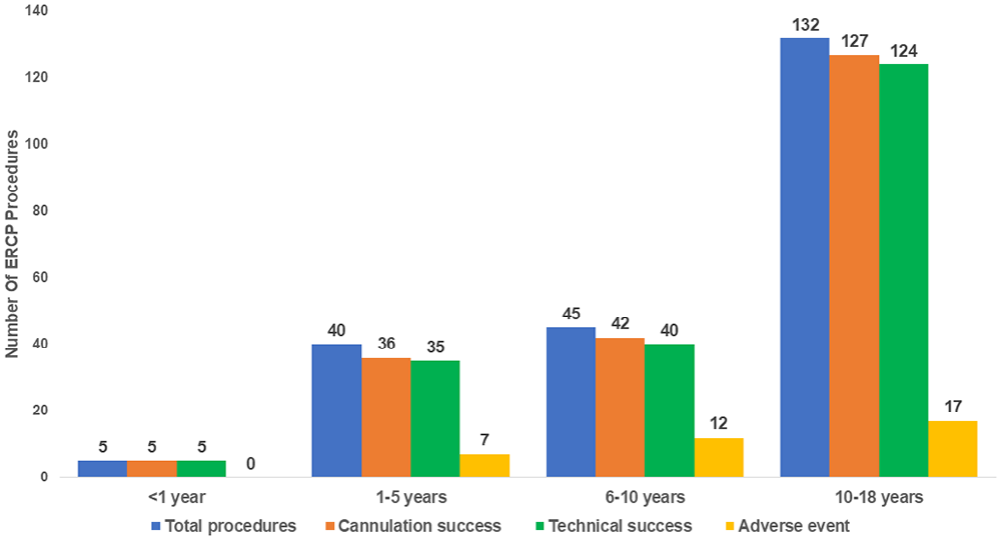
Age‐group‐wise cannulation and technical success rate of all endoscopic retrograde cholangiopancreatography (ERCP) procedures.

Among 222 children undergoing ERCP, cannulation and technical success rates were 210 (94.5%) and 204 (92%), respectively. The cannulation and technical success rates of all the ERCP procedures were comparable in all age groups (Table 2 and Figure 2).

Among the ERCP procedures, a total of 36 (16%) adverse events occurred with no mortality (Table 3). None of the infants had any procedure‐related adverse events. Age‐group‐wise distribution of adverse events is depicted in Figure 2. Adverse event rates were comparable across all age groups (*p* = 0.52). PEP (18, 8%) was the most common adverse event, followed by post‐sphincterotomy bleeding (12, 5.4%, 10 minor, two major). The majority (15, 83%) of cases of PEP were mild while three (17%) were moderately severe with two requiring percutaneous drainage. Among the 10 children with minor post‐sphincterotomy bleeding, 8 stopped spontaneously while the remaining two cases were managed with injection adrenaline (1:10,000 dilution). In the two cases of major bleeding, hemostasis was achieved using ‘Coagrasper Hemostatic Forceps’ (Olympus Inc.) using the electrosurgical unit ERBE VIO 300D (setting, SOFT COAG mode; effect, 5; max watts, 60; ERBE).

**TABLE 3:** Adverse events across various age groups in children undergoing endoscopic retrograde cholangiopancreatography procedures.

	<1 year (*n*, %)	1–5 years (*n*, %)	5–10 years (*n*, %)	10–18 years (*n*, %)	All age groups (*n*, %)	*p‐*value^#^
Total patients	5	40	45	132	222	–
Total procedures	5	57	62	162	286	–
Post‐sphincterotomy bleeding						
Minor	0 (0)	2 (5)	3 (6.6)	5 (3.8)	10 (4.5)	0.79
Major	0 (0)	1 (2.5)	0 (0)	1 (0.7)	2 (1)	0.88
Post‐ERCP pancreatitis	0 (0)	2 (5) [two mild]	7 (15) [six mild and one moderately severe]	9 (7) [seven mild and two moderately severe]	18 (8)	0.15
Cholangitis	0 (0)	0 (0)	0 (0)	1 (0.7)	1 (0.4)	0.75
Suspected perforation	0 (0)	0 (0)	1 (2)	0 (0)	1 (0.4)	0.61
Stent migration						
External	0 (0)	1(2.5)	0 (0)	1 (0.7)	2 (1)	0.87
Internal	0 (0)	0 (0)	0 (0)	0 (0)	0 (0)	0.88
Stent breakage	0 (0)	0 (0)	0 (0)	0 (0)	0 (0)	–
Sedation‐related complications	0 (0)	1 (2.5)	1 (2)	0 (0)	2 (1)	0.53
Total	0 (0)	7 (17.5)	12 (26)	17 (13)	36 (16)	0.73

All the procedural outcomes were analyzed based on based on patient‐based numbers.

^#^ *p*‐value is calculated for particular adverse events across age groups 1–5, 5–10, and 10–18 years as no adverse event occurred in children <1‐year group.

The indications, procedural details, and adverse events of endoscopic cystogastrostomy are summarized in Table 4. The commonest indication of endoscopic cystogastrostomy was acute pancreatitis with fluid collection (87.5%; pseudocyst 53%, walled‐off necrosis 34.5%) while seven (12.5%) cases were performed in children with chronic pancreatitis with pseudocyst. The cannulation and technical success rates were both 96%. There were two adverse events (3.5%), one minor bleeding (managed conservatively), and another internal stent migration in the pseudocyst cavity. In the case of internal migration of double pigtail plastic stent into the pancreatic pseudocyst, the stent was retrieved endoscopically with rat‐tooth forceps after dilating the same fistulous tract of cystogastrostomy.

**TABLE 4:** Indications and procedural details of endoscopic cystogastrostomy procedures in children across various age groups.

	<1 year (*n*, %)	1–5 years (*n*, %)	5–10 years (*n*, %)	10–18 years (*n*, %)	All age groups (*n*, %)	*p‐*value^#^
Total patients	0	4	10	41	55	–
Total procedures	0	5	11	41	57	–
Median age in years, (IQR)	–	4 (4–4.5)	8 (5.5–9)	13 (11.5–15.5)	11 years (IQR 4–14)	–
Gender, Male	0	2	6	24	32	
Median body weight in kg (IQR)	–	13 (11–14)	24.5 (19–27)	41 (34–43.5)	36 (20.5–41)	–
Indications:						
Acute pancreatitis with pseudocyst		4 (100)	5 (50)	20 (49)	29 (53)	–
Acute pancreatitis with WON		0 (0)	3 (30)	16 (39)	19 (34.5)	–
Chronic pancreatitis with pseudocyst		0 (0)	2 (20)	5 (12)	7 (12.5)	0.71
Endoscopic cystogastrostomy						
Plastic stent	0 (0)	4 (100)	10 (100)	41 (100)	55 (100)	0.999
Metallic stent	0 (0)	0 (0)	0 (0)	0 (0)	0 (0)	–
Cannulation success rate	–	3/4 (75)	9/10 (90)	41/41 (100)	53/55 (96)	0.89
Technical success rate	–	3/4 (75)	9/10 (90)	41/41(100)	53/55 (96)	0.89
**Adverse events**	0 (0)	0 (0)	2 (20)	0 (0)	2 (3.5)	0.68
Bleeding						
Minor	0 (0)	0 (0)	1(10)	0 (0)	1 (1.7)	0.07
Major	0 (0)	0 (0)	0 (0)	0 (0)	0 (0)	–
Stent migration						
External	0 (0)	0 (0)	0 (0)	0 (0)	0 (0)	–
Internal	0 (0)	0 (0)	1(10)	0 (0)	1 (1.7)	0.065
Stent breakage	0 (0)	0 (0)	0 (0)	0 (0)	0 (0)	–
Sedation‐related complication	0 (0)	0 (0)	0 (0)	0 (0)	0 (0)	–

## DISCUSSION

In one of the largest series of ERCP in children, we have shown that ERCP can be effectively and safely performed even in the under‐five children, using standard adult endoscopes and accessories by pediatric gastroenterologists with high cannulation (95%) and technical success rates (92%) and acceptable adverse event rate (16%).

Even though the etiological spectrum and outcome in children vary from the adults, the current understanding and approach to the management of various hepato‐pancreatico‐biliary diseases are mostly based on adult studies, owing to the sparse published pediatric literature.^3^, ^4^, ^5^, ^6^, ^7^, ^8^, ^9^, ^10^, ^11^, ^12^, ^18^, ^19^, ^20^, ^21^ ERCP is often underutilized in pediatric populations as it is technically demanding, and requires a greater amount of training and skills. Lack of experience, as well as the uncertainty of its efficacy, could be the reasons behind its underutilization in children. Most previous pediatric ERCP studies were conducted in Western populations,^3^, ^4^, ^5^, ^6^, ^7^, ^8^, ^18^, ^19^, ^20^, ^21^ and data from the rest of the world are limited.^9^, ^10^, ^11^, ^12^ Prior studies from India included predominantly older children^9^, ^10^, ^11^, ^12^ and shared the experience of either biliary^9^ or pancreatic procedures.^11^ The study by Poddar et al., reported their experience of 84 pediatric ERCP procedures due to various biliary (52%) and pancreatic disorders (48%); however, only 26% of them were therapeutic procedures.^10^ Dahale et al., shared their experience of 164 ERCPs among Indian children; however, only 13 (8%) of them were for under‐five children.^12^ In our study, we shared the decade‐long experience of 286 ERCP procedures in 222 children which is the second‐largest series of pediatric ERCP worldwide and the largest from a Pediatric Gastroenterology center. A considerable proportion (20%) of ERCPs in our study were performed in under‐five children. The largest single‐center series so far is from the Czech Republic and from a busy adult gastroenterology center.^7^ Table 5 summarizes the study population, indications, success rate, and adverse events of various studies reporting ERCP in under‐five children as well as the current study.

**TABLE 5:** Various pediatric studies with the details of endoscopic retrograde cholangiopancreatography procedures.

	Felux et al.^18^	Limketkai et al.^19^	Keil et al.^7^	Avitsland et al.^21^	Dahale et al.^10^	Our study
Total number of children	31	154	624	158	127	222
<5 years of age	14	33	349 (<6 years)	58	13	45
No. of ERCP sessions	54	289	402	244	164	286
Indications						
Biliary	85%	56%	92%	18%	54%	66%
Pancreatic	15%	44%	8%	82%	46%	34%
Therapeutic	88%	72%	58%	90%	67%	94%
Diagnostic	12%	28%	42%	10%	33%	6%
Cannulation success rate	90.7%	94%	94%	88%	90.4%	94.5%
Technical success rate	90.7%	91%	92%	86%	86%	92%
Adverse events PEP Cholangitis Bleeding Perforation Stent migration Anesthesia‐related complications	5 (9.3%) 4 (mild) 1 0 0 0 0	17 (6%) 12 (10 mild and two moderate) 0 2 0 0 3	3 (0.7%) 1 (mild) 1 0 1 0 0	9 (6%) 8 (seven mild and one moderate) 0 1 0 2 0	8 (4.8%) 2 (mild) 0 2 2 0 2	7 (15%) 2 (mild) 0 3 0 1 1
Mortality	0		0	0	0	0

Abbreviations: ERCP, endoscopic retrograde cholangiopancreatography; PEP, post‐ERCP pancreatitis.

ERCP has different utilities for different age groups. In infants, it is still mainly a diagnostic procedure to confirm or exclude biliary atresia, like infants in our study.^19^ Although it is not routinely recommended as part of the diagnostic evaluation of neonatal cholestasis, it may be beneficial in selected patient groups and may obviate exploratory laparotomy in such patients. Kiel et al,^7^ and Limketkai et al,^20^ reported the indications of pediatric ERCPs across all age groups, like our study. They have shown that in children below 5 years of age, the main indication for ERCP was biliary diseases (choledochal cyst followed by choledocholithiasis), similar to our results. In contrast, older children underwent ERCP predominantly due to choledocholithiasis and pancreatic diseases, like our study.^7^, ^20^

Most of the previous pediatric ERCP studies have reported cannulation and technical success rates to the tune of 90%, comparable to our results, which highlight the efficacy and feasibility of ERCP in the pediatric population.^10^, ^12^, ^18^, ^19^, ^20^, ^21^ The study by Kiel et al., reported a technical success rate of 76% in infants, 92% in children between 1–6 years of age, and 94% in children >6 years of age.^7^ On the contrary, our results showed that all the five ERCPs performed in infants were successful. However, the technical success rates were similar in older age groups, comparable to the study by Keil et al.^7^ The fact that many (13%) of the ERCPs done in infants in the study by Kiel et al., were therapeutic, in contrast to none in our study, could be the reason for the higher success rate of ERCP in infants in our study.

ERCP is not without its adverse events. A recent systematic review and meta‐analysis on pediatric ERCP has shown a pooled complication rate of 6% with PEP (4.7%) being the most common, followed by bleeding (0.6%), and infections (0.8%).^22^ The study by Kiel et al., reported a complications rate of 9.35% with PEP (7%) being the most common, followed by bleeding (0.4%) and cholangitis (0.4%), similar to our findings.^7^ Children are theoretically at a higher risk of developing PEP than adults due to the smaller size of the papilla relative to standard cannulation equipment.^23^ Previous studies have reported a PEP incidence of 3%–17% in children undergoing ERCP due to any indications.^7^, ^18^, ^19^, ^20^, ^24^, ^25^ The PEP rates (5.5%) in our study were comparable to those reported for both adults^26^, ^27^ and children.^21^, ^22^, ^23^, ^24^ Other complications such as cholangitis, stent migration, and suspected perforation were less in number and were managed conservatively with no mortality, comparable to other studies.^7^, ^19^, ^20^, ^21^, ^22^ Anaesthesia‐related complications were encountered in only 0.6% of cases, comparable to other pediatric studies, supporting the fact that deep sedation can be safely utilized in children.^7^, ^10^, ^12^, ^19^, ^20^, ^21^, ^22^

Endoscopic cystogastrostomy is the preferred method of drainage of bulging pancreatic fluid collections in children.^28^ However, pediatric literature is limited, especially in under‐five children.^29^, ^30^ The systematic review and meta‐analysis of 14 pediatric studies by Nabi et al. demonstrated that endoscopic cystogastrostomy is a safe and effective method of pancreatic fluid drainage, similar to our findings.^28^

Our study has certain limitations. First, our study was retrospective in nature and conducted at a single tertiary care hospital. Hence, there could be a possibility of potential selection bias and the results are not generalizable. Secondly, long‐term follow‐up was not available, which is especially more relevant to endotherapy in the setting of chronic calcific pancreatitis. Furthermore, risk factors for adverse events associated with the procedures could not be analyzed due to their small number.

## CONCLUSIONS

The results of our study suggested that ERCP is feasible, effective, and can be safely performed even in under‐five children, using standard adult duodenoscopes (diagnostic scope for <3 years and therapeutic scope for 3–5 years) and accessories.

## CONFLICT OF INTEREST STATEMENT

None.

## ETHICS STATEMENT

The study was performed in a manner to conform with the Helsinki Declaration of 1975, as revised in 2000 and 2008, concerning Human and Animal Rights.

## PATIENT CONSENT STATEMENT

Informed consent was obtained from either parent before all procedures. The Institutional Ethical Committee (IEC) has given approval for the study (IEC 2024‐163‐IP‐EXP‐60).

## CLINICAL TRIAL REGISTRATION

N/A.

## References

[1] McCune WS , Shorb PE , Moscowitz H . Endoscopic cannulation of the ampulla of Vater: A preliminary report. Ann Surg 1968; 167: 752. 5646296 10.1097/00000658-196805000-00013 PMC1387128

[2] Waye JD . Endoscopic retrograde cholangiopancreatography in the infant. Am J Gastroenterol 1976; 65: 461–3. 949055

[3] Varadarajulu S , Wilcox CM , Hawes RH , Cotton PB . Technical outcomes and complications of ERCP in children. Gastrointest Endosc 2004; 60: 367–7. 15332025 10.1016/s0016-5107(04)01721-3

[4] Cheng CL , Fogel EL , Sherman S *et al*. Diagnostic and therapeutic endoscopic retrograde cholangiopancreatography in children: A large series report. J Pediatr Gastroenterol Nutr 2005; 41: 445–53. 16205513 10.1097/01.mpg.0000177311.81071.13

[5] Pant C , Sferra TJ , Barth BA *et al*. Trends in endoscopic retrograde cholangiopancreatography in children within the United States, 2000–2009. J Pediatr Gastroenterol Nutr 2014; 59: 57–60. 24509307 10.1097/MPG.0000000000000333

[6] Giefer MJ , Kozarek RA . Technical outcomes and complications of pediatric ERCP. Surg Endosc 2015; 29: 3543–50. 25673350 10.1007/s00464-015-4105-1

[7] Keil R , Drábek J , Lochmannová J *et al*. ERCP in infants, children, and adolescents–Different roles of the methods in different age groups. PLoS One 2019; 14: e0210805. 30653580 10.1371/journal.pone.0210805 PMC6336232

[8] Makita S , Amano H , Kawashima H *et al*. Utility of endoscopic retrograde cholangiopancreatography in management of pediatric pancreaticobiliary disease. BMC Pediatr 2022; 22: 134. 35287648 10.1186/s12887-022-03207-3 PMC8919614

[9] Sharma AK , Wakhlu A , Sharma SS . The role of endoscopic retrograde cholangiopancreatography in the management of choledochal cysts in children. J Pediatr Surg 1995; 30: 65–7. 7722833 10.1016/0022-3468(95)90612-6

[10] Poddar U , Thapa BR , Bhasin DK , Prasad A , Nagi B , Singh K . Endoscopic retrograde cholangiopancreaticography in the management of pancreaticobiliary disorders in children. J Gastroenterol Hepatol 2001; 16: 927–31. 11555109 10.1046/j.1440-1746.2001.02545.x

[11] Agarwal J , Nageshwar Reddy D , Talukdar R *et al*. ERCP in the management of pancreatic diseases in children. Gastrointest Endosc 2014; 79: 271–8. 24060520 10.1016/j.gie.2013.07.060

[12] Dahale AS , Puri AS , Sachdeva S , Srivastava S , Kumar A . Endoscopic retrograde cholangiopancreaticography in children: A single‐center experience from Northern India. Indian Pediatr 2019; 56: 196–8. 30954989

[13] Dumonceau JM , Delhaye M , Tringali A *et al*. Endoscopic treatment of chronic pancreatitis: European Society of Gastrointestinal Endoscopy (ESGE) Guideline – Updated August 2018. Endoscopy 2019; 51: 179–93. 30654394 10.1055/a-0822-0832

[14] Dumonceau JM , Andriulli A , Elmunzer BJ *et al*. Prophylaxis of post‐ERCP pancreatitis: European Society of Gastrointestinal Endoscopy (ESGE) Guideline – Updated June 2014. Endoscopy 2014; 46: 799–815. 25148137 10.1055/s-0034-1377875

[15] ASGE Standards of Practice Committee , Chandrasekhara V , Khashab MA *et al*. Adverse events associated with ERCP. Gastrointest Endosc 2017; 85: 32–47. 27546389 10.1016/j.gie.2016.06.051

[16] Cotton PB , Eisen GM , Aabakken L *et al*. A lexicon for endoscopic adverse events: Report of an ASGE workshop. Gastrointest Endosc 2010; 71: 446–54. 20189503 10.1016/j.gie.2009.10.027

[17] Banks PA , Bollen TL , Dervenis C *et al*. Acute Pancreatitis Classification Working Group. Classification of acute pancreatitis–2012: Revision of the Atlanta classification and definitions by international consensus. Gut 2013; 62: 102–11. 23100216 10.1136/gutjnl-2012-302779

[18] Uc A , Husain SZ . Pancreatitis in children. Gastroenterology 2019; 156: 1969–78. 30716320 10.1053/j.gastro.2018.12.043 PMC6730664

[19] Felux J , Sturm E , Busch A *et al*. ERCP in infants, children and adolescents is feasible and safe: Results from a tertiary care center. United European Gastroenterol J 2017; 5: 1024–9. 10.1177/2050640616687868 PMC5676540 29163969

[20] Limketkai BN , Chandrasekhara V , Kalloo AN . Okolo PI 3rd. Comparison of performance and safety of endoscopic retrograde cholangiopancreatography across pediatric age groups. Dig Dis Sci 2013; 58: 2653–60. 23709156 10.1007/s10620-013-2691-0

[21] Avitsland TL , Aabakken L . Endoscopic retrograde cholangiopancreatography in infants and children. Endosc Int Open 2021; 9: E292–6. 33655024 10.1055/a-1337-2212 PMC7892276

[22] Usatin D , Fernandes M , Allen IE , Perito ER , Ostroff J , Heyman MB . Complications of endoscopic retrograde cholangiopancreatography in pediatric patients; A systematic literature review and meta‐analysis. J Pediatr 2016; 179: 160–5. 27663215 10.1016/j.jpeds.2016.08.046 PMC5123955

[23] Otto AK , Neal MD , Slivka AN , Kane TD . An appraisal of endoscopic retrograde cholangiopancreatography (ERCP) for pancreaticobiliary disease in children: Our institutional experience in 231 cases. Surg Endosc 2011; 25: 2536–40. 21359895 10.1007/s00464-011-1582-8

[24] Troendle DM , Abraham O , Huang R , Barth BA . Factors associated with post‐ERCP pancreatitis and the effect of pancreatic duct stenting in a pediatric population. Gastrointest Endosc 2015; 81: 1408–16. 25686874 10.1016/j.gie.2014.11.022

[25] Saito T , Terui K , Mitsunaga T *et al*. Role of pediatric endoscopic retrograde cholangiopancreatography in an era stressing less‐invasive imaging modalities. J Pediatr Gastroenterol Nutr 2014; 59: 204–9. 24762457 10.1097/MPG.0000000000000399

[26] Chen JJ , Wang XM , Liu XQ *et al*. Risk factors for post‐ERCP pancreatitis: A systematic review of clinical trials with a large sample size in the past 10 years. Eur J Med Res 2014; 19: 26. 24886445 10.1186/2047-783X-19-26 PMC4035895

[27] ASGE Standards of Practice Committee , Buxbaum JL , Freeman M *et al*. American Society for Gastrointestinal Endoscopy guideline on post‐ERCP pancreatitis prevention strategies: Methodology and review of evidence. Gastrointest Endosc 2023; 97: 163–183.e40. 36517309 10.1016/j.gie.2022.09.011

[28] Nabi Z , Talukdar R , Lakhtakia S , Reddy DN . Outcomes of endoscopic drainage in children with pancreatic fluid collections: A systematic review and meta‐analysis. Pediatr Gastroenterol Hepatol Nutr 2022; 25: 251–62. 35611379 10.5223/pghn.2022.25.3.251 PMC9110851

[29] Poddar U , Yachha SK , Upadhyaya VD *et al*. Endoscopic cystogastrostomy: Still a viable option in children with symptomatic pancreatic fluid collection. Pancreatology 2021; 21: 812–8. 33602644 10.1016/j.pan.2021.02.004

[30] Sharma SS , Maharshi S . Endoscopic management of pancreatic pseudocyst in children‐a long‐term follow‐up. J Pediatr Surg 2008; 43: 1636–9. 18778998 10.1016/j.jpedsurg.2008.01.026

